# Serum-Free Manufacturing of Mesenchymal Stem Cell Tissue Rings Using Human-Induced Pluripotent Stem Cells

**DOI:** 10.1155/2019/5654324

**Published:** 2019-01-15

**Authors:** Tackla S. Winston, Kantaphon Suddhapas, Chenyan Wang, Rafael Ramos, Pranav Soman, Zhen Ma

**Affiliations:** ^1^Department of Biomedical & Chemical Engineering, Syracuse University, Syracuse NY 13244, USA; ^2^Syracuse Biomaterials Institute, Syracuse University, Syracuse NY 13244, USA

## Abstract

Combination of stem cell technology and 3D biofabrication approaches provides physiological similarity to *in vivo* tissues and the capability of repairing and regenerating damaged human tissues. Mesenchymal stem cells (MSCs) have been widely used for regenerative medicine applications because of their immunosuppressive properties and multipotent potentials. To obtain large amount of high-quality MSCs without patient donation and invasive procedures, we differentiated MSCs from human-induced pluripotent stem cells (hiPSC-MSCs) using serum-free E6 media supplemented with only one growth factor (bFGF) and two small molecules (SB431542 and CHIR99021). The differentiated cells showed a high expression of common MSC-specific surface markers (CD90, CD73, CD105, CD106, CD146, and CD166) and a high potency for osteogenic and chondrogenic differentiation. With these cells, we have been able to manufacture MSC tissue rings with high consistency and robustness in pluronic-coated reusable PDMS devices. The MSC tissue rings were characterized based on inner diameter and outer ring diameter and observed cell-type-dependent tissue contraction induced by cell-matrix interaction. Our approach of simplified hiPSC-MSC differentiation, modular fabrication procedure, and serum-free culture conditions has a great potential for scalable manufacturing of MSC tissue rings for different regenerative medicine applications.

## 1. Introduction

Tissue engineering has offered a promising treatment option for tissue repair and regeneration, as it is intended to produce a biomimetic construct similar to the native tissue and to restore physiological functions [1]. Specifically, ring-shaped tissue constructs have been engineered for vascular and trachea tissue engineering purposes for better structural mimicry. Based on the self-assembly of smooth muscle cells (SMCs), engineered vascular tissue rings have been fabricated in the agarose wells with SmGM-2 media supplemented with 20% FBS, PDGF, and TGF-*β*1 growth factors [2]. In addition, hybrid fibroblast-MSC tissue rings were fabricated with collagen scaffolds and fused to form a tubular structure for trachea tissue engineering [3]. Tissue rings offer even force distribution across the constructs. This feature circumvents the concentration of forces in the center that is often observed in conventional sheet-shaped tissue constructs. Ring shapes are also easier to handle and suture to the damaged native tissues with standard clinical procedures.

Current tissue ring engineering still heavily relies on primary cells, especially MSCs from bone marrow or adipose tissues [4]. Harvesting primary MSCs requires an invasive and painful procedure that yields only about 0.001%–0.01% from bone marrow and about 0.05% from liposuction [5, 6]. In addition, MSCs show reduction in both quantity and quality from older individuals, who are the primary population requiring autologous MSCs for disease treatment. Moreover, isolated MSCs have shown limited differentiation potential and reduced viability after several passages [7, 8]. To overcome these limitations, current MSC research has been steered towards the derivation of MSCs from human-induced pluripotent stem cells (hiPSC-MSCs). Early attempts for hiPSC-MSC differentiation involved either murine feeder layer or embryoid body formation [9–13]. More recent approaches used cocktails of growth factors and small molecules to generate hiPSC-MSCs in a more controlled way [9]. However, most methods are still depended on serum-containing culture media. High content of xenogeneic proteins in animal serum that might provoke an immune reaction in patients is one of the primary concerns for tissue engineering and cell therapeutic applications. High degree of batch-to-batch variation of serum production would also cause inconsistency in the generation of quality-assured cells, which makes the standardization of the cell and tissue manufacturing process very difficult.

In this study, we developed a simplified serum-free MSC differentiation protocol using two small molecules, SB431542 and CHIR99021, which inhibit TGF-*β* and GSK-3*β* pathways, respectively. Our differentiation protocol generated defined MSC population by controlling their developmental trajectory through intermediate neural crest stem cells (NCSCs). We successfully differentiated hiPSC-MSCs and used these cells to create mechanically viable tissue rings. Based on the morphological features of these tissue rings, we investigated how different fabrication and culture conditions would affect the ring quality. Taking advantage of a robust fabrication procedure and serum-free culture condition, we were able to fabricate the MSC tissue ring with high consistency and still maintain the multipotent MSC characteristics.

## 2. Materials and Methods

### 2.1. hiPSC-MSC Differentiation and Maintenance

The hiPSC line (WTC) was established in Bruce R. Conklin lab at Gladstone Institute of Cardiovascular Disease by reprogramming from adult skin fibroblasts from an Asian male. The hiPSCs were maintained on 6-well plates coated with Geltrex reduced growth factor (Life Technologies, Ca# A1413302) in Essential 8 (E8) media (Life Technologies, Ca# A1517001). Geltrex diluted for thin coating was prepared by thawing 5 mL of original Geltrex gel into 495 mL cold DMEM/F12 (Life Technologies, Ca# 11320033). hiPSCs were plated at a density of 2.5 × 10^4^ cells/cm^2^ onto the Geltrex coated 6-well plates in E8 with 10 *μ*M Y-27632 (BioVision, Ca# 1784). hiPSCs were maintained in E8 for two additional days and then induced for differentiation at “day 0” with differentiation media that consists of 10 ng/mL bFGF (R&D Systems, Ca# 233-FB), 4 *μ*M SB431542 (Stemgent, Ca# 04-0010-10), and 4 *μ*M WNT agonist CHIR99021 (CHIR) (Stemgent, 04-2004) in Essential 6 (E6) media (Life Technologies, A1516401). Differentiation media were changed daily for the next 5 days. On day 6, the differentiated NCSCs were plated as “multipotent passage 0” (MP0) on a Geltrex coated 6-well plate in serum-free MSC culture media (CTS StemPro MSC SFM) (Life Technologies, A1033201) at a density of 4.0 × 10^4^ cells/cm^2^. Every 6 days, the cells are replated at a density of 2.0 × 10^4^ cells/cm^2^ for MP1 and then 1.0 × 10^4^ cells/cm^2^ for MP2–MP10. Starting from MP3, the surface coating switched from Geltrex to 1% gelatin to support hiPSC-MSC adhesion and growth. MP5–MP10 hiPSC-MSCs were used to generate the tissue rings.

### 2.2. Flow Cytometry

The hiPSC-MSC differentiation was characterized using flow cytometry. Cells were singularized with 0.25% trypsin for 5 minutes and quenched with serum-free media. After washing with DPBS three times, cells were fixed with 4% (vol/vol) paraformaldehyde (PFA) for 15 minutes and incubated with fluorescent conjugated antibodies against cell surface markers: CD105, CD90, CD146, CD45, and CD73 (Human MSC Multi-Color Flow Kit, R&D Systems, Ca# FMC002) for 45 minutes each in wash/permeabilization buffer (R&D Systems, Ca# FC005). The labeled cells were analyzed by the BD Accuri™ C6 flow cytometer in Syracuse University Flow Core.

### 2.3. Immunocytochemistry

Cells were fixed with 4% (vol/vol) PFA for 15 minutes and blocked with 2% BSA for 30 minutes. Cells were incubated with primary antibodies (Supplementary Table 1) for 2 hours at room temperature or overnight at 4°C and then incubated with secondary antibodies for 1.5 hours at room temperature. DAPI was used to stain cell nuclei. For bright-field and epifluorescence microscopy, the images were taken using a Nikon Eclipse Ti microscope with Zyla 4.2 PLUS sCMOS camera.

### 2.4. Histology

Tissue rings were fixed with 4% PFA overnight at 4°C and dehydrated with 30% sucrose solution (Fisher Scientific, S5-500) for 3 hours. Next, tissue rings were embedded with optimal cutting temperature compound (Fisher Scientific, 50363579), frozen overnight at −80°C, and then sectioned to a thickness of 10 *μ*m with Cryostat (Sakura, 6203) in McDonald lab at Syracuse University. H&E staining was performed with the standard protocol. Tissue rings were rehydrated by alcohol with gradient concentration and counterstained with hematoxylin (Leica, 3801560) and eosin (Leica, 3801600). After dehydration with alcohol, tissue rings were cleared in xylene (Sigma-Aldrich, XX0060-4) and mounted with Permount (Fisher Scientific, SP15-100) on glass slides. Images were taken with the Leica ICC50 microscope.

### 2.5. Trilineage Differentiation

The hiPSC-MSC trilineage differentiation and characterization were performed using human MSC functional identification kit (R&D Systems, SC006). To induce osteogenic differentiation, hiPSC-MSCs were plated on a sterilized coverslip in a 24-well plate at 4.2 × 10^3^ cells/cm^2^ and allowed to attach overnight or until 50–70% confluent. Osteogenic medium consisting of *α*MEM (Life Technologies, Ca# 12561056) and osteogenic supplement was replaced twice weekly. After 21 days, cells were incubated with anti-osteocalcin primary antibody overnight at 4°C and then with secondary antibody. For adipogenic induction, hiPSC-MSCs were plated on a sterilized coverslip in a 24-well plate at 2.1 × 10^4^ cells/cm^2^ and allowed to grow until 100% confluent. Adipogenic medium consisting of *α*MEM and adipogenic supplement was replaced twice weekly. After 21 days, FABP4 was used to detect lipid droplets. To induce chondrogenesis, 2.5 × 10^5^ hiPSC-MSCs were transferred to a 15 mL falcon tube and pelleted by centrifugation (1000 rpm for 5 minutes). The supernatant was removed, and chondrogenic differentiation medium consisting of DMEM/F-12, ITS supplement, and chondrogenic supplement was changed every 3 days. After 21 days, pellets were sectioned and stained with anti-aggrecan antibody for chondrocytes. Samples were visualized using the Nikon Eclipse Ti microscope with Zyla 4.2 PLUS sCMOS camera.

### 2.6. Mold Fabrication

The acrylonitrile butadiene styrene (ABS) molds-negatives were designed using SolidWorks, converted into *.stl* files, and printed using the Zortrax M200 high-resolution extrusion 3D printer with 0.19 mm layer thickness. These ABS molds were designed and fabricated for casting the polydimethylsiloxane (PDMS) devices, each of which had a center post (2 mm in diameter) and a trough (3 mm in width) for the formation of tissue rings. Entire device can fit in a cell culture well (21 mm in diameter) of the regular 24-well plate, which potentially offers easy handling for larger production of tissue rings. To facilitate the PDMS device removal from the ABS molds, we coated the molds with silicone spray (Stoner, G0093241). PDMS base and curing agent were mixed at a ratio of 10 : 1 (*w*/*w*) in weighing boat and poured into the molds. The molds containing PDMS were then transferred into a desiccator, thoroughly degassed for about 3 hours to remove all bubbles, and cured in an oven at 60°C overnight. The PDMS devices were then cut out using the Xacto knife from the ABS molds, rinsed in 70% ethanol for 15 minutes, and stored at room temperature.

Pluronic acid solution was used to coat PDMS devices to achieve a nonfouling surface to reduce cell adhesion to the PDMS surface. 10% pluronic acid was made by adding 1 g of Pluronic® F-127 (Sigma-Aldrich, P2443) in 10 mL distilled water and dissolved in the oven at 50°C overnight. The 10% pluronic acid was sterilized using the Steriflip® filter unit (0.22 *μ*m, EMD Millipore) and stored at room temperature. One day before cell seeding, the PDMS devices were rinsed with 70% ethanol and Dulbecco's phosphate-buffered saline (DPBS) (Life Technologies, Ca# 14190144) consecutively and then incubated with 10% pluronic acid for 24 hours at room temperature.

### 2.7. Tissue Ring Formation and Extraction

hiPSC-MSCs were rinsed with DPBS, incubated with trypsin (Life Technologies, Ca# 25200056) for 5 minutes at 37°C and quenched with cell culture media. The cell suspension was collected in a 15 mL tube, centrifuged to a pellet, and resuspended in the desired cell culture media. Cell number was determined using a hemocytometer. Collagen scaffold was prepared by mixing rat tail collagen I (Life Technologies, A1048301), 1 N NaOH (Sigma-Aldrich, 1091371000), distilled water, and 10x PBS (Life Technologies, AM9625) based on the manufacturer's instruction. Since collagen would rapidly gel at room temperature, other reagents were mixed in advance before adding collagen. When the cell suspension was ready, collagen I and cell suspension were added into the premixed solutions to achieve different collagen densities (0.5 mg/mL, 1 mg/mL, and 2 mg/mL) with different total cell numbers (0.3 million cells and 0.6 million cells). These procedures should be completed on ice to avoid collagen being clogged inside the prechilled pipette tips. The cell-collagen mixture was loaded into PDMS devices and incubated at 37°C with 5% CO_2_ for 40 minutes, before cell culture media were filled to the top of the devices. The hiPSC-MSC tissue rings were cultured with different media, 5% serum media (DMEM supplemented with FBS), 10% serum media (DMEM supplemented with FBS), and serum-free media (CTS StemPro MSC SFM). The media were changed every two days.

hiPSC-MSC tissue rings were formed within 24 hours after cell seeding into the PDMS devices. Tissue rings could then be extracted out of the PDMS device after about 3 days or when strong enough to be gently pushed up to the top of the center posts. The morphology of tissue rings was imaged and examined using the Nikon Eclipse Ti microscope with the Zyla 4.2 PLUS sCMOS camera and NIS Element software. Each tissue ring was measured at least three times stochastically for inner diameter and outer ring diameter. The measurements were then averaged for each tissue ring.

## 3. Results and Discussion

**Figure 1:**
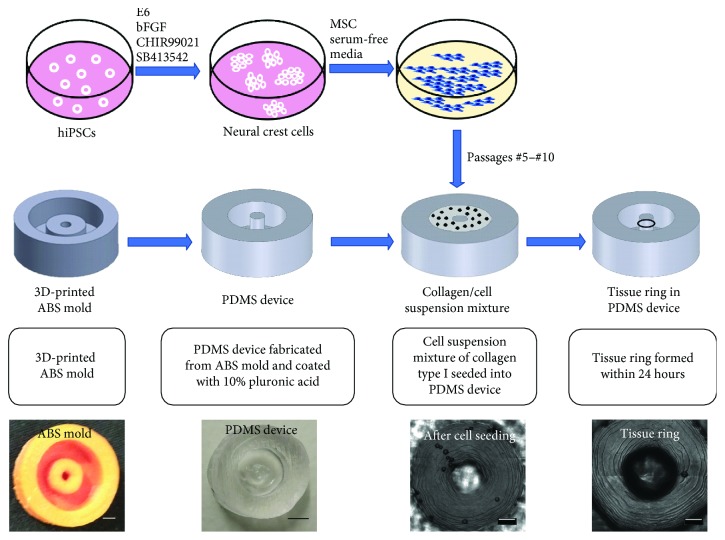
Fabrication of hiPSC-MSC tissue rings. The hiPSCs were differentiated into NCSCs, which were then further differentiated into MSCs using a serum-free chemical-defined protocol. The PDMS devices were cast from 3D-printed ABS molds and coated with 10% pluronic acid before cell seeding. Passage #5–#10 hiPSC-MSCs were mixed with collagen scaffold and loaded into the PDMS devices to form the tissue rings surrounding the center posts. Scale bar: 1 mm.

We derived our MSCs from hiPSCs using chemical-defined media without any serum supplement (Figure S1). We have been able to passage our hiPSC-MSCs up to MP17 and still observed MSC-like spindle morphology and high expression of MSC surface markers. We have optimized the process for fabricating hiPSC-MSC tissue rings (Figure 1). We printed the ABS molds (Figure S2) using the Zortrax M200 high-resolution extrusion 3D printer with a 0.19 mm layer thickness and cast the PDMS devices with a 2 mm diameter center post. Starting from passage #5 (MP5), hiPSC-MSCs were used to fabricate the tissue rings by mixing with collagen scaffold in the PDMS devices coated with 10% pluronic acid. Within 24 hours after seeding cells into the devices, cells aggregated surrounding the center post and formed the tissue rings.

### 3.1. Derivation of Functional MSCs from hiPSCs

**Figure 2:**
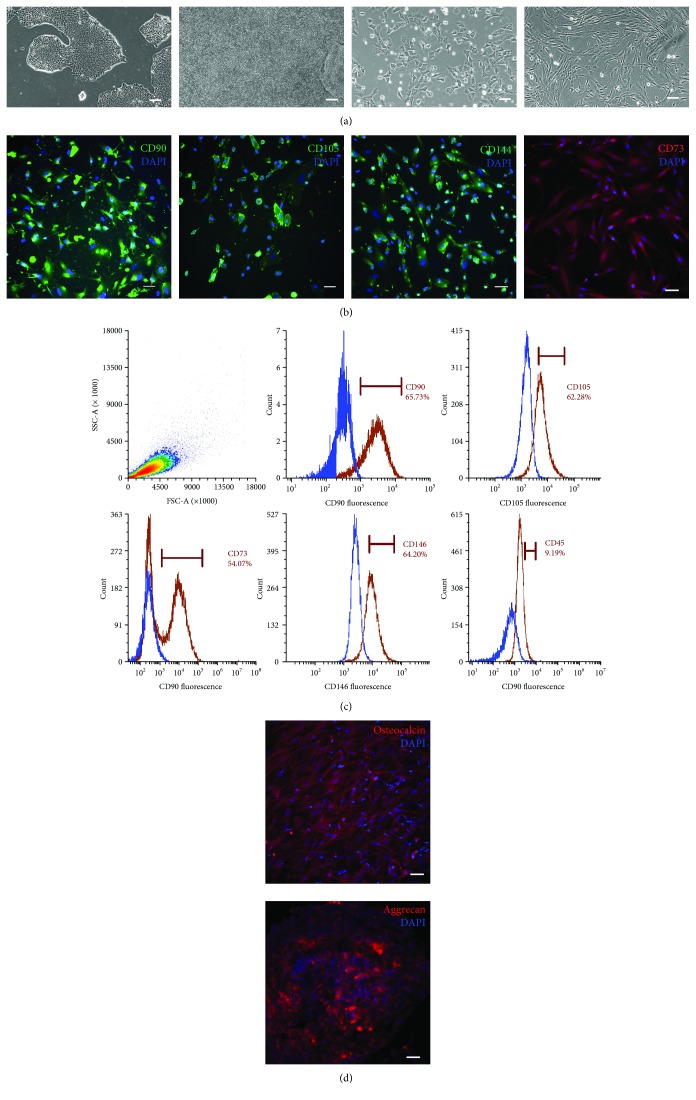
Characterization of hiPSC-MSCs. (a) The morphological changes during hiPSC-MSC differentiation from hiPSC colonies, dense NCSCs in differentiation, dissociated single NCSCs, and spindle-shaped hiPSC-MSCs at passage #4 (MP4). (b) hiPSC-MSCs expressed CD90, CD105, CD144, and CD73, confirmed by immunocytochemistry. (c) Flow cytometry histograms showed that hiPSC-MSCs were positive for CD90, CD105, CD73, and CD146 and negative for CD45 starting at MP4. (d) The hiPSC-MSCs also demonstrated the ability for osteogenic and chondrogenic differentiation. Scale bar: (a) 100 *μ*m, (b, c) 50 *μ*m.

The hiPSCs were first differentiated into neural crest stem cells (NCSCs) using E6 media supplemented with 10 ng/mL bFGF, 4 *μ*M CHIR, and 4 *μ*M SB. To confirm successful NCSC differentiation, we performed the immunofluorescent staining and showed that intermediate cells expressed NCSC markers (SOX10, FOX3D, and NGFR) cultured in the differentiation medium (Figure S3(a)). The NCSCs were then transferred into serum-free MSC culture media for further differentiation into MSCs. After four passages in the serum-free media, cells changed from the round-like morphology to the spindle-like morphology (Figure 2(a)). Immunofluorescences confirmed that our hiPSC-MSCs expressed the MSC-specific surface protein markers that were commonly used to characterize primary hMSCs, such as CD90, CD73, CD105, and CD144 (Figure 2(b)). Further analysis of the immunophenotype of the hiPSC-MSCs was performed using flow cytometry at MP4, which showed positive expression of mesenchymal markers CD73, CD90, CD105, and CD146, but low expression of the hematopoietic marker CD45 (Figure 2(c)). We also directly seeded our hiPSC-MSCs to the cell culture dish without any protein coating. We observed that the hiPSC-MSCs were able to adhere and proliferate on plain plastic. We also performed immunofluorescent staining on our hiPSC-MSCs grown on plain plastic and found that they highly expressed the markers of CD44, CD106, and CD166 (Figure S3(b)). We also confirmed that intermediate NCSCs and hiPSC-MSCs had no expression of pluripotent markers NANOG (Figure S3(c)).

The hiPSC-MSCs were then subjected to trilineage differentiation. The hiPSC-MSCs displayed robust osteogenic and chondrogenic differentiation as indicated by osteocalcin and aggrecan staining, respectively (Figure 2(d)). However, our hiPSC-MSCs showed limited adipogenic differentiation with little evidence of lipid droplet accumulation. Unlike primary bone marrow or adipose tissue-derived hMSCs that is inclined to adipogenic differentiation by default, hiPSC-MSCs derived from the developmental route of NCSCs had a greater propensity to undergo osteogenic and chondrogenic differentiation rather than adipogenesis. Similar results were also reported that hiPSC-MSCs demonstrated robust differentiation into osteocytes and chondrocytes, but limited differentiation into adipocytes [5].

### 3.2. Robust Tissue Ring Fabrication

**Figure 3:**
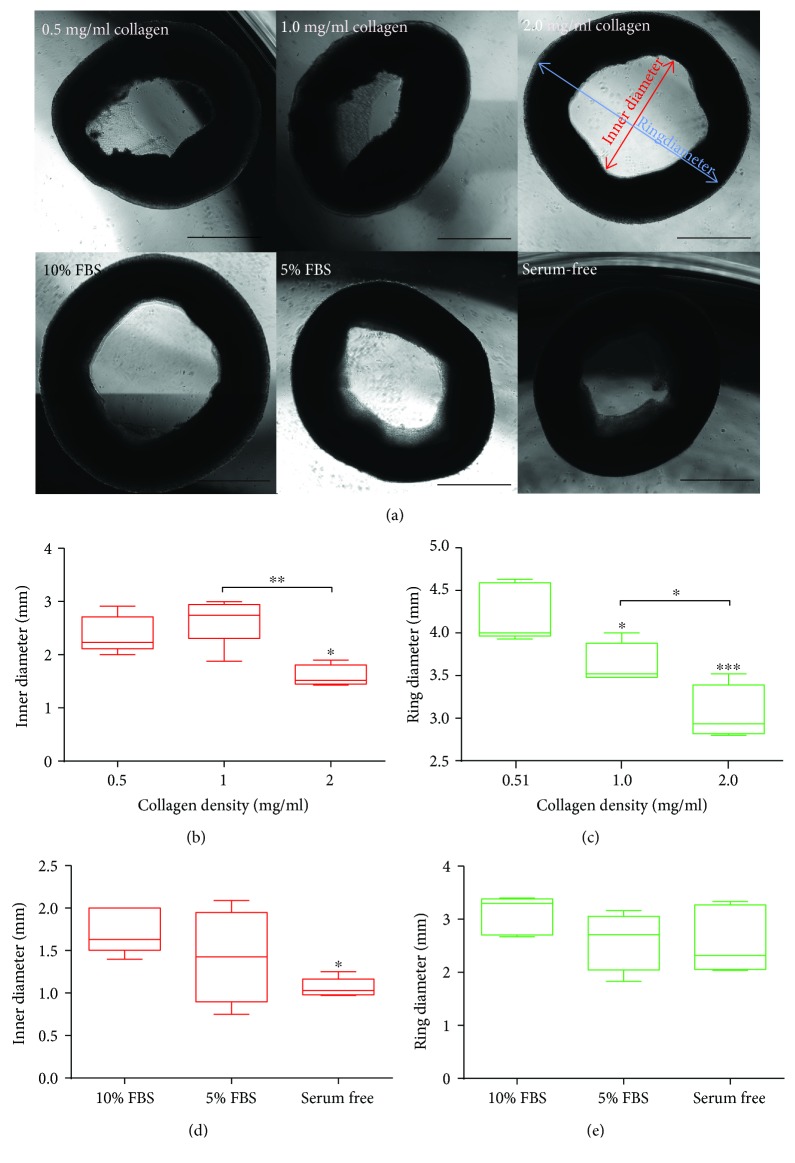
Characterization of hiPSC-MSC tissue rings. (a) The shape of hiPSC-MSC tissue rings 24 hours after extraction fabricated with different collagen densities and different culture media. (b) hiPSC-MSC tissue rings had smaller inner diameter at 2 mg/mL collagen density compared to lower densities. (c) The ring diameter decreased with increase in collagen density. hiPSC-MSC tissue rings showed (d) smaller inner diameter and (e) comparable ring diameter, for serum-containing media and serum-free media. Scale bar: 1 mm.

We successfully generated hiPSC-MSC rings (0.6 million cells) at different collagen densities (0.5 mg/mL, 1 mg/mL, and 2 mg/mL) and in different cell culture media (10% serum media, 5% serum media, and serum-free media). Within 24 hours after extraction of hiPSC-MSC tissue ring from PDMS devices, we characterized two primary geometrical features (inner diameter and outer ring diameter) for the different conditions (Figure 3(a)). We found that hiPSC-MSC tissue rings with 2 mg/mL collagen density had significantly smaller inner diameter than those with lower collagen density (Figure 3(b)), indicating that high collagen content may induce tissue contraction. Similarly, the ring diameter decreased with the increase in collagen density, suggesting that collagen increased the remodeling rate of the tissue rings (Figure 3(c)). Tissue contraction mediated by collagen scaffold has been well studied in free-floating collagen model [14, 15]. Fibroblast-laden collagen scaffolds were consistently observed with contraction over time, indicating that cell-matrix interaction leads to the tissue remodeling process. Particularly, in our tissue ring model, we observed that high collagen density would induce rapid tissue contraction after the rings were extracted from the PDMS devices.

Serum-based media remain a common standard culture condition in generating and expanding hMSCs. Previous reports on the tissue rings engineered from hMSCs and SMCs were heavily dependent on serum-containing media. In our study, we derived our hiPSC-MSCs with a serum-free approach and explored whether we could also generate hiPSC-MSC tissue rings without the serum supplements. With different cell culture media, we found that hiPSC-MSC tissue rings cultured with serum-free media showed smaller inner diameter compared to those with serum-containing media (Figure 3(d)); however, there was no statistical difference in ring diameter among the different cell culture media (Figure 3(e)). As far as we know, our results indicate the first-time tissue rings have been robustly produced from hiPSC-MSCs in serum-free and chemically defined media.

**Figure 4:**
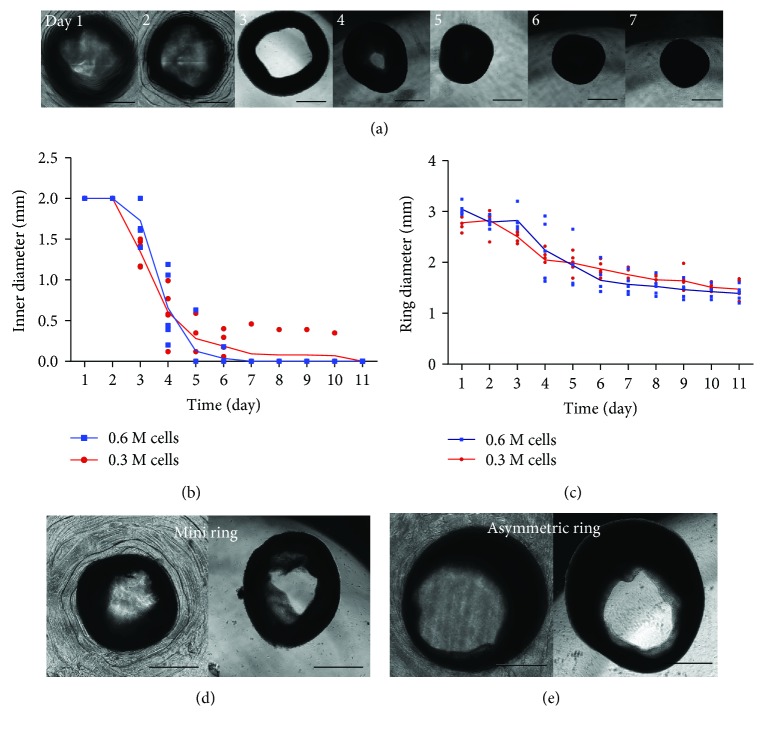
Morphological changes of hiPSC-MSC tissue rings. (a) The morphology of hiPSC-MSC tissue rings before and after extraction indicated that ring shape turned into circular shape during continuous culturing. The tissue rings produced with low cell number (0.3M cells in total) showed no significant difference in (b) inner diameter and (c) ring diameter compared to standard cell number (0.6M cells in total). (d) The mini tissue rings and (e) asymmetric tissue rings were also fabricated with different designs of PDMS devices. Scale bar: 1 mm.

Interestingly, the hiPSC-MSC tissue rings exhibited morphological changes after they were extracted out of the PDMS devices (Figure 4(a)). The inner diameter decreased and finally closed up at around three days after extraction, which was not seen on the tissue rings fabricated with human fibroblasts (Figure S4). These results indicated that the tissue rings fabricated with different cell types exhibited different morphogenic behaviors, which might be related to the growth kinetics of specific cell types. We found that our hiPSC-MSCs had a very high proliferation rate, especially when they were cultured with serum-free media. It has been reported that interleukin (IL) 4 and 13 promoted MSC-laden collagen gel contraction [16]. Active secretion of cytokines and growth factors from hiPSC-MSCs would also lead to the dynamic morphological changes of ring shapes that were not observed from fibroblast tissue rings.

To increase the manufacturing efficiency, we decreased the total cell number used for each tissue ring from 0.6 million (0.6M) to 0.3 million (0.3M) and found no significant difference on both inner diameter and ring diameter between the two conditions (Figures 4(b) and 4(c)). Even with a low cell number, we still found that hiPSC-MSC tissue rings turned into a circular shape after extraction out of the PDMS devices. This demonstrates an increase in the throughput of tissue ring production using minimal total cell number and shows a great potential in scale-up tissue manufacturing with limited stem cell sources. We also explored the fabrication of mini tissue rings using small PDMS devices (fit in a 48-well cell culture plate) and asymmetric tissue rings using the PDMS devices with off center posts (Figure S2). We have been able to create and extract mini tissue rings with ~2 mm ring diameter (Figure 4(d)) and asymmetric tissue rings with wider tissue on one side compared to the other side (Figure 4(e)). This shows that the method can be used to fabricate tissue rings of different sizes and shapes with high mechanical integrity for different applications.

### 3.3. Chondrogenic Differentiation of hiPSC-MSC Tissue Rings

**Figure 5:**
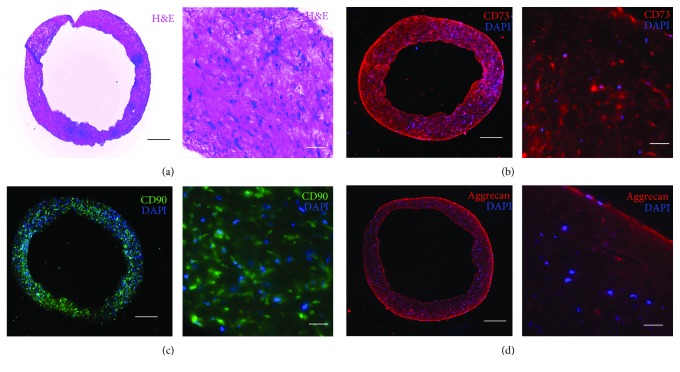
Histochemistry of hiPSC-MSC tissue rings. (a) H&E staining of hiPSC-MSC tissue rings represented cytoplasm (pink) and nuclei (blue). Immunofluorescent staining of hiPSC-MSC tissue ring with (b) CD73 and (c) CD90 demonstrated that the tissue rings maintained the MSC biomarkers after the fabrication process. (d) The hiPSC-MSC tissue rings have been successfully differentiated into chondrogenic lineage, indicating that the tissue rings retained the potency for further differentiation. Scale bar: whole tissue structure (left) 500 *μ*m and zoom-in close-ups (right) 10 *μ*m.

To further characterize our hiPSC-MSC tissue rings, we performed hematoxylin and eosin (H&E) staining, which showed a highly cellularized tissue ring (Figure 5(a)). Via immunohistochemical analysis, we confirmed that our hiPSC-MSCs continually maintained MSC characteristics after being engineered into a tissue ring by the expression of CD90 and CD73 (Figures 5(b) and 5(c)). In addition, we successfully induced chondrogenic differentiation on the hiPSC-MSC tissue rings. Biomaterials and tissue engineering approaches have been studied to develop tracheal substitutes for the treatment of narrowing or collapsing trachea. A functional trachea consists of cartilage rings capable of supporting an open windpipe. Previous studies have shown that primary hMSC tissue rings could be differentiated into cartilaginous rings and tubes by adding TGF-*β*1-loaded microspheres for trachea tissue engineering. In our system, hiPSC-MSCs formed the ring shape 24 hours after seeding with collagen scaffold and differentiation could be induced after day 3. We confirmed our chondrogenic differentiation by aggrecan staining, which showed a uniform expression across the tissue rings (Figure 5(d)). We found that the chondrogenic tissue rings could maintain the same inner diameter after removal from the PDMS devices, indicating that morphological changes of the ring shape were highly cell-type dependent. Our approach could potentially eliminate the need for microspheres as the carriers of additional growth factors to engineer cartilaginous rings from hiPSC-MSCs.

hiPSC-MSCs offer distinct advantages over primary MSCs, such as consistent production in large quantities, improvement of differentiation potentials, and relative homogeneous cell populations, which are amenable to the scale-up manufacturing process for “off-shelf” or “on-demand” cell products [17–19]. Hence, our simplified protocol of hiPSC-MSC differentiation could be essential for biomanufacturing applications. On the other hand, engineering and manufacturing biologically functional tissues still face great challenges in scalable production, quality maintenance, and tissue transportation to patients, especially for complex tissue constructs. Our ring-shaped design offers a structured but simple tissue construct that could potentially be used in most commercially available bioreactors; thus, our method could be scaled up to a clinically effective, reproducible, and safe process. By combining with robotics, mold fabrication and tissue assembly could be achieved in a more controlled and automated fashion, towards the goal of tissue manufacturing for regenerative medicine.

## 4. Discussions

Over the past decade, the protocols for direct differentiation of MSC from ESCs or hiPSCs have been continuously improved. Earliest attempts on MSC differentiation were completed on feeder cells by directly seeding the ESCs in the medium containing 20% FBS that allowed the cells to spontaneously differentiate from pluripotent state to multipotent state [20]. After 40 days, cell population only resulted in around 5% CD73+ MSC-like cells, which were then sorted based on the CD73+ surface marker [20]. The cells were then expanded for 7–14 days with several passages till cells showed a spindle-like morphology [20]. Few years later, an embryonic body- (EB-) based approach was developed for MSC differentiation through the formation of EBs on a gelatin-coated plate with 10% FBS-supplemented medium [21, 22]. Outgrowing cells that migrated out of the EBs were collected and cultured for 2 weeks, till they showed a spindle-like morphology. These cells showed a high expression of MSC surface markers, such as CD90, CD73, CD105, and CD166, and a low expression of hematopoietic stem cell (HSC) surface markers, such as CD34, CD45, and CD14. These cells were able to achieve adipogenic, osteogenic, and chondrogenic differentiation. By reducing the serum content, knockout serum replacement was used with the supplementation of bFGF to differentiate monolayer hiPSCs into MSCs [23]. The cells had a high expression of CD29+, CD44+, CD73+, CD90+, and CD105+ surface markers and a low expression of CD11b−, CD14−, CD31−, and CD34− surface markers. These hiPSC-MSCs showed efficient osteogenic and chondrogenic differentiation, but very limited adipogenic differentiation compared to bone marrow-derived MSCs.

The use of SB431542 for inhibiting the TGF-*β* signaling pathway has been widely adapted for MSC differentiation from hPSCs [5, 24, 25]. Mahmood et al. [24] differentiated MSCs from EBs with knockout serum replacement and SB431542 in the absence of bFGF and then maintained them in serum-containing medium (10% FBS). ESC-MSCs showed a positive expression of CD44, CD73, CD146, and CD166 and a negative expression of CD34 and CD56, in addition to adipogenic and osteogenic differentiation, but no chondrogenic differentiation was performed. Adapting a similar protocol, Sánchez et al. [25] differentiated MSCs from monolayer hESCs in the medium supplemented with TGF-*β*1 and SB431542. hESC-MSCs were sorted for CD73+/CD90+ expression and maintained with serum-containing medium (10% FBS). Trilineage differentiation showed efficient osteogenic and chondrogenic differentiation, but relatively low adipogenic differentiation. Chen et al. [5] derived MSCs from monolayer ESCs and hiPSCs by inhibiting the TGF-*β* signaling pathway with SB431542 for 10 days in knockout serum medium. The hPSC-MSCs showed a positive expression of MSC surface markers (CD73, CD90, and CD105) and a negative expression of HSC surface markers (CD117, CD34, and CD45). Like many other studies, hPSC-MSCs in this study exhibited robust osteogenic and chondrogenic differentiation, whereas limited adipogenic differentiation. These were consistent with other research that has shown that primary deciduoplacental MSCs [10], umbilical cord blood [11], Wharton's jelly [12, 13], and fetal MSCs [14, 15] have limited adipogenicity compared to adult bone marrow MSCs.

Recently, there has been a shift in the field of hiPSC-MSC differentiation to better standardize the MSC differentiation process by controlling their developmental trajectory with defined intermediate cell types. The study conducted by Eto et al. [26] derived hiPSC-MSCs based on two distinct developmental processes, mesodermal and neuroepithelial differentiation, based on different combinations of growth factors in serum-containing medium (mesoderm: 10% FBS plus bFGF, BMP4, activin, and LiCl; neuroepithelial: 10% FBS plus 100 nM retinoic acid). The MSCs were sorted based on PDGFR+/VEGFR2+ cells. The purified cells were characterized based on MSC surface markers and trilineage differentiation and showed no significant difference between the hiPSC-MSCs derived from two intermediate cell types.

In development, NCSCs undergo epithelial-mesenchymal transition from neuroepithelium, migrate to the mesoderm layer, and give rise to both ectodermal lineages (neural cells and pigment cells) and mesenchymal lineages (cranial bone and cartilage, primary aorta, and cardiac cushions) [27]. All these tissue types have limited presence of adipose tissue at early development. When we differentiated NCSCs to mesenchymal derivatives, our MSCs showed a high potential to differentiate into bone, cartilage, and vascular cells. It has even been hypothesized that dental pulp MSCs are probably derived from NCSCs. This motivates us to control the differentiation pathway to an intermediate cell type such as NCSCs, which might enable us to obtain better defined MSC population compared to other existing approaches. We took the advantage of the NCSC differentiation protocol developed by Menendez et al. [28], who used CHIR99021 in combination with SB431542 in a serum-based culture system. Hence, our new approach is able to not only derive hiPSC-MSCs under serum-free culture conditions but also generate well-defined MSC population by controlling their developmental trajectory through NCSCs as intermediate cell type.

Among all the tissue constructs, ring-shaped tissue constructs offer several advantages over conventional sheet-shaped tissue constructs: (1) tissue rings offer even force distribution across the tissues, since the ring-shaped design circumvents concentration of internal force in the center [29], (2) tissue rings are easier to handle and suture to damaged native tissues with standard clinical procedures [30], and (3) specifically for cardiovascular tissue, tissue rings could potentially be used to mimic the structural feature of vascular tissues. A stack assembly of tissue rings could be used to emulate special tissue architecture, such as vasculature and trachea, by mimicking the cross section of these tissues for better understanding their physiological and mechanical functions.

Generation of tissue rings for vascular tissue engineering has been pursued based on either SMCs or MSCs. Engineered vascular tissue rings were created in agarose molds from both rat SMCs (rSMCs) and human SMCs (hSMCs) with high mechanical integrity [31]. By performing tensile strength testing, it was found that rSMC tissue rings were structurally thicker and mechanically stronger than hSMC tissue rings after 14 days of culture. In addition, the tissue rings generated from self-assembled human primary bone marrow MSCs (hMSCs) lacked mechanical strength and failed in the mechanical testing. Vascular tissue rings have also been generated with hiPSC-derived SMCs (hiPSC-SMCs) using culture medium with 20% FBS, PDGF-BB, and TGF-*β*1 growth factors [2]. Low FBS medium (5% FBS) failed to generate tissue rings with the hiPSC-SMCs. Contractility of hiPSC-SMC tissue rings was determined for drug evaluation and disease phenotyping. Carbachol and KCl were found to increase the contractility of the tissue rings. hiPSC-SMC tissue rings generated from patients with supravalvular aortic stenosis showed reduced responsiveness to the carbachol, compared to the healthy control hiPSC-SMC tissue rings. For trachea tissue engineering [32], hMSC-based cartilaginous tissue rings were generated to create tissue tubes by stacking multiple rings together. To increase the tissue thickness and mechanical strength, TGF*β*-loaded microspheres were mixed with the hMSCs for tissue ring fabrication. hMSC + microsphere tissue tubes were found to have similar tensile strength compared to the native rat trachea.

Although we emphasized the cartilage tissue engineering in our tissue ring study, we believe our hiPSC-MSCs will have a broader impact on the research related to MSC-based cell therapies, especially for bone and vascular tissue engineering. Human primary MSCs have demonstrated their superior capability of immunomodulation by the secretion of a variety of growth factors and cytokines [33, 34], which put MSCs at the frontier of clinical trials. For example, secretome analysis of MSCs indicated that IGFBP7 produced by human MSCs would promote the osteogenesis for bone repair [35]. A series of studies have also suggested that the secretion of TFG-*α*, VEGF, and PDGF from MSCs would promote angiogenesis *in vitro* and *in vivo*. In the future, we will perform secretome analysis on our hiPSC-MSCs, pursuing a new way of regenerative medicine based on cell-free therapeutic strategies [36].

## 5. Conclusions

To conclude, we have been able to establish a robust protocol to induce the conversion of hiPSCs into MSC-like cells under serum-free culture condition that eliminated the variabilities resulting from animal serum production. Our hiPSC-MSCs showed a high expression of MSC-specific biomarkers and had the capability of osteogenic and chondrogenic differentiation. We also demonstrated that these cells can be used in a modular system to engineer MSC tissue rings with minimal cell density and high morphological consistency. We were able to differentiate the MSC tissue rings into cartilaginous tissues, which could potentially be used for trachea tissue engineering.

## Data Availability

The authors declare that all data supporting the findings of this study are available within the paper and its supplementary information. Source data for the figures are available from the corresponding author upon reasonable request.

## Supplementary Materials

Supplementary Materials Supplementary Figure 1: the differentiation protocol for hiPSC-MSCs through intermediate cell type of NCSCs. Supplementary Figure 2: SolidWorks designs for the molds that were used to fabricate standard tissue rings and asymmetrical tissue rings in a 24-well plate and mini tissue rings in a 48-well plate. Supplementary Figure 3: (a) intermediate cells expressed NCSC-specific markers of SOX10, FOXD3, and NGFR; (b) hiPSC-MSCs expressed MSC-specific surface markers of CD44, CD106, and CD166; (c) hiPSCs showed a high expression of NANOG, but NCSCs and hiPSC-MSCs showed no expression of NANOG. Scale bar: 50 *μ*m. Supplementary Figure 4: morphological changes of fibroblast tissue rings. (a) The morphology of fibroblast tissue rings after extraction retained the ring shape with consistent (b) outer ring diameter and (c) inner diameter. Scale bar: 1 mm. Supplementary Table 1: the primary antibodies used for immunofluorescence microscopy in this study.

## References

[1] Flanagan T. C., Pandit A. (2003). Living artificial heart valve alternatives: a review. *European Cells and Materials*.

[2] Dash B. C., Levi K., Schwan J. (2016). Tissue-engineered vascular rings from human iPSC-derived smooth muscle cells. *Stem Cell Reports*.

[3] Naito H., Tojo T., Kimura M. (2011). Engineering bioartificial tracheal tissue using hybrid fibroblast-mesenchymal stem cell cultures in collagen hydrogels. *Interactive Cardiovascular and Thoracic Surgery*.

[4] Caplan A. I., Correa D. (2011). The MSC: an injury drugstore. *Cell Stem Cell*.

[5] Chen Y. S., Pelekanos R. A., Ellis R. L., Horne R., Wolvetang E. J., Fisk N. M. (2012). Small molecule mesengenic induction of human induced pluripotent stem cells to generate mesenchymal stem/stromal cells. *Stem Cells Translational Medicine*.

[6] Kern S., Eichler H., Stoeve J., Klüter H., Bieback K. (2006). Comparative analysis of mesenchymal stem cells from bone marrow, umbilical cord blood, or adipose tissue. *Stem Cells*.

[7] Stolzing A., Jones E., McGonagle D., Scutt A. (2008). Age-related changes in human bone marrow-derived mesenchymal stem cells: consequences for cell therapies. *Mechanisms of Ageing and Development*.

[8] Zhou S., Greenberger J. S., Epperly M. W. (2008). Age-related intrinsic changes in human bone-marrow-derived mesenchymal stem cells and their differentiation to osteoblasts. *Aging Cell*.

[9] Fu Q. L., Chow Y. Y., Sun S. J. (2012). Mesenchymal stem cells derived from human induced pluripotent stem cells modulate T-cell phenotypes in allergic rhinitis. *Allergy*.

[10] Barlow S., Brooke G., Chatterjee K. (2008). Comparison of human placenta-and bone marrow–derived multipotent mesenchymal stem cells. *Stem Cells and Development*.

[11] Rebelatto C. K., Aguiar A. M., Moretão M. P. (2008). Dissimilar differentiation of mesenchymal stem cells from bone marrow, umbilical cord blood, and adipose tissue. *Experimental Biology and Medicine*.

[12] Hsieh J.-Y., Fu Y.-S., Chang S.-J., Tsuang Y.-H., Wang H.-W. (2010). Functional module analysis reveals differential osteogenic and stemness potentials in human mesenchymal stem cells from bone marrow and Wharton’s jelly of umbilical cord. *Stem Cells and Development*.

[13] Kim M. J., Shin K. S., Jeon J. H. (2011). Human chorionic-plate-derived mesenchymal stem cells and Wharton’s jelly-derived mesenchymal stem cells: a comparative analysis of their potential as placenta-derived stem cells. *Cell and Tissue Research*.

[14] Ngo P., Ramalingam P., Phillips J. A., Furuta G. T. (2006). Collagen gel contraction assay. *Cell-Cell Interactions in Health and Disease*.

[15] Feng Z., Yamato M., Akutsu T., Nakamura T., Okano T., Umezu M. (2003). Investigation on the mechanical properties of contracted collagen gels as a scaffold for tissue engineering. *Artificial Organs*.

[16] Liu X., Kohyama T., Wang H. (2002). Th2 cytokine regulation of type I collagen gel contraction mediated by human lung mesenchymal cells. *American Journal of Physiology-Lung Cellular and Molecular Physiology*.

[17] Langer R. S., Vacanti J. P. (1999). Tissue engineering: the challenges ahead. *Scientific American*.

[18] Sipe J. D. (2002). Tissue engineering and reparative medicine. *Annals of the New York Academy of Sciences*.

[19] Naughton G. K. (2002). From lab bench to market. *Annals of the New York Academy of Sciences*.

[20] Barberi T., Willis L. M., Socci N. D., Studer L. (2005). Derivation of multipotent mesenchymal precursors from human embryonic stem cells. *PLoS Medicine*.

[21] Brown S. E., Tong W., Krebsbach P. H. (2008). The derivation of mesenchymal stem cells from human embryonic stem cells. *Cells Tissues Organs*.

[22] Hwang N. S., Varghese S., Lee H. J. (2008). In vivo commitment and functional tissue regeneration using human embryonic stem cell-derived mesenchymal cells. *Proceedings of the National Academy of Sciences of the United States of America*.

[23] Kang R., Zhou Y., Tan S. (2015). Mesenchymal stem cells derived from human induced pluripotent stem cells retain adequate osteogenicity and chondrogenicity but less adipogenicity. *Stem Cell Research & Therapy*.

[24] Mahmood A., Harkness L., Schrøder H. D., Abdallah B. M., Kassem M. (2010). Enhanced differentiation of human embryonic stem cells to mesenchymal progenitors by inhibition of TGF-*β*/activin/nodal signaling using SB-431542. *Journal of Bone and Mineral Research*.

[25] Sánchez L., Gutierrez-Aranda I., Ligero G. (2011). Enrichment of human ESC-derived multipotent mesenchymal stem cells with immunosuppressive and anti-inflammatory properties capable to protect against experimental inflammatory bowel disease. *Stem Cells*.

[26] Eto S., Goto M., Soga M. (2018). Mesenchymal stem cells derived from human iPS cells via mesoderm and neuroepithelium have different features and therapeutic potentials. *PLoS One*.

[27] Achilleos A., Trainor P. A. (2012). Neural crest stem cells: discovery, properties and potential for therapy. *Cell Research*.

[28] Menendez L., Kulik M. J., Page A. T. (2013). Directed differentiation of human pluripotent cells to neural crest stem cells. *Nature Protocols*.

[29] Shazly T., Rachev A., Lessner S. (2015). On the uniaxial ring test of tissue engineered constructs. *Experimental Mechanics*.

[30] Strobel H. A., Calamari E. L., Alphonse B., Hookway T. A., Rolle M. W. (2018). Fabrication of custom agarose wells for cell seeding and tissue ring self-assembly using 3D-printed molds. *Journal of Visualized Experiments*.

[31] Gwyther T. A., Hu J. Z., Billiar K. L., Rolle M. W. (2011). Directed cellular self-assembly to fabricate cell-derived tissue rings for biomechanical analysis and tissue engineering. *Journal of Visualized Experiments*.

[32] Dikina A. D., Strobel H. A., Lai B. P., Rolle M. W., Alsberg E. (2015). Engineered cartilaginous tubes for tracheal tissue replacement via self-assembly and fusion of human mesenchymal stem cell constructs. *Biomaterials*.

[33] Tögel F. E., Westenfelder C. (2010). Mesenchymal stem cells: a new therapeutic tool for AKI. *Nature Reviews. Nephrology*.

[34] Monsel A., Zhu Y. G., Gennai S., Hao Q., Liu J., Lee J. W. (2014). Cell-based therapy for acute organ injury. *Anesthesiology*.

[35] Infante A., Rodríguez C. I. (2018). Secretome analysis of in vitro aged human mesenchymal stem cells reveals IGFBP7 as a putative factor for promoting osteogenesis. *Scientific Reports*.

[36] Vizoso F., Eiro N., Cid S., Schneider J., Perez-Fernandez R. (2017). Mesenchymal stem cell secretome: toward cell-free therapeutic strategies in regenerative medicine. *International Journal of Molecular Sciences*.

