# Supplementary material for: Serum-Free Manufacturing of Mesenchymal Stem Cell Tissue Rings Using Human-Induced Pluripotent Stem Cells
Source: Stem Cells Int. 2019 Jan 15;2019:5654324. doi: 10.1155/2019/5654324 (PMC6350554; doi:10.1155/2019/5654324)
Supplement: Supplementary Materials — Supplementary Figure 1: the differentiation protocol for hiPSC-MSCs through intermediate cell type of NCSCs. Supplementary Figure 2: SolidWorks designs for the molds that were used to fabricate standard tissue rings and asymmetrical tissue rings in a 24-well plate and mini tissue rings in a 48-well plate. Supplementary Figure 3: (a) intermediate cells expressed NCSC-specific markers of SOX10, FOXD3, and NGFR; (b) hiPSC-MSCs expressed MSC-specific surface markers of CD44, CD106, and CD166; (c) hiPSCs showed a high expression of NANOG, but NCSCs and hiPSC-MSCs showed no expression of NANOG. Scale bar: 50 μm. Supplementary Figure 4: morphological changes of fibroblast tissue rings. (a) The morphology of fibroblast tissue rings after extraction retained the ring shape with consistent (b) outer ring diameter and (c) inner diameter. Scale bar: 1 mm. Supplementary Table 1: the primary antibodies used for immunofluorescence microscopy in this study. [file 5654324.f1.pdf]

SUPPLEMENTARY MATERIALS:

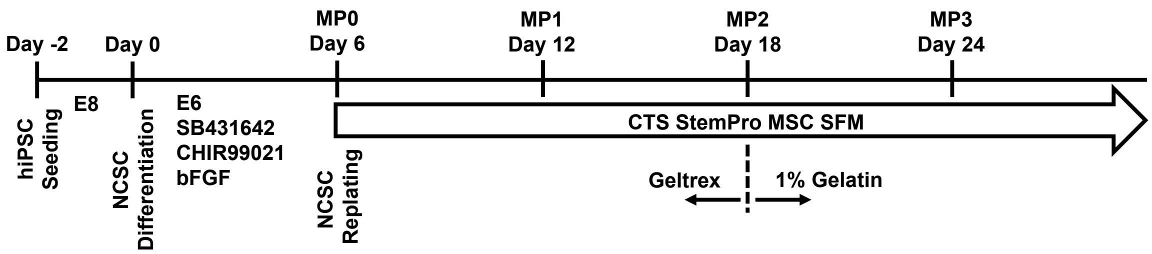

Supplementary Figure 1: The differentiation protocol for hiPSC-MSCs through intermediate cell type of NCSCs.

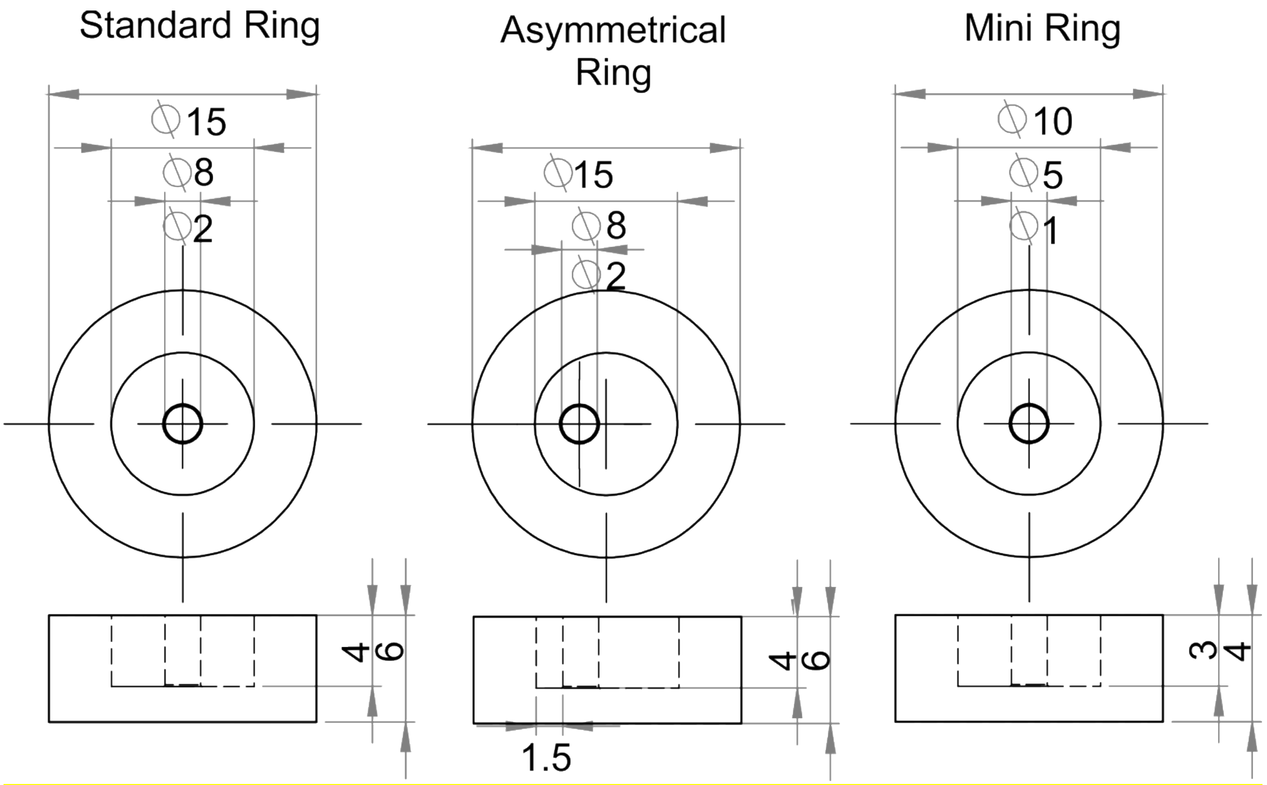

Supplementary Figure 2: SolidWork designs for the molds that were used to fabricate standard tissue rings and asymmetrical tissue rings in 24-well plate, and mini tissue rings in 48-well plate.

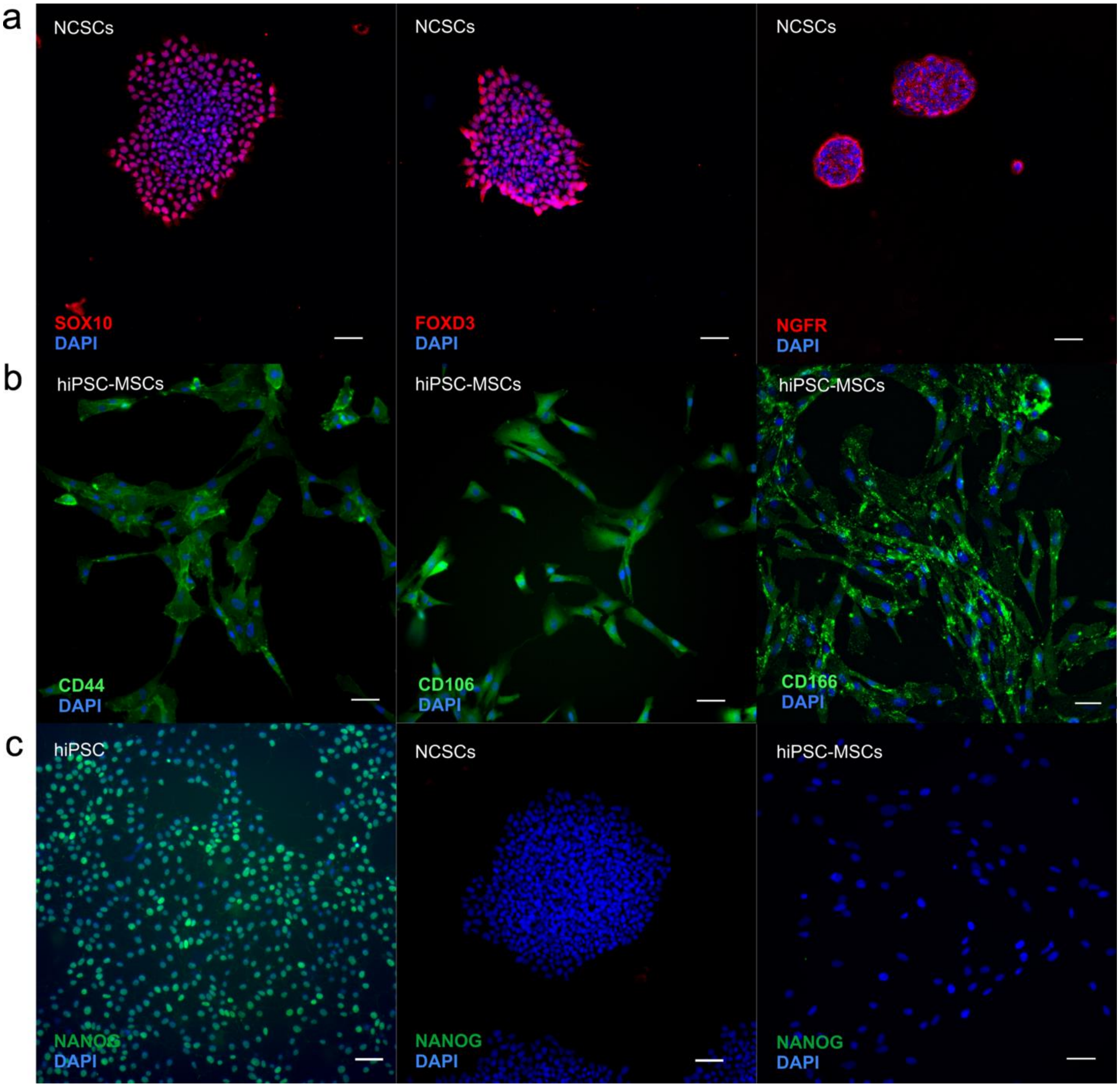

Supplementary Figure 3: (a) Intermediate cells expressed NCSC-specific markers of SOX10, FOXD3 and NGFR. (b) hiPSC-MSCs expressed MSC-specific surface markers of CD44, CD106 and CD166. (c) hiPSCs showed high expression of NANOG, but NCSCs and hiPSC-MSCs showed no expression of NANOG. Scale bar: 50 µm.

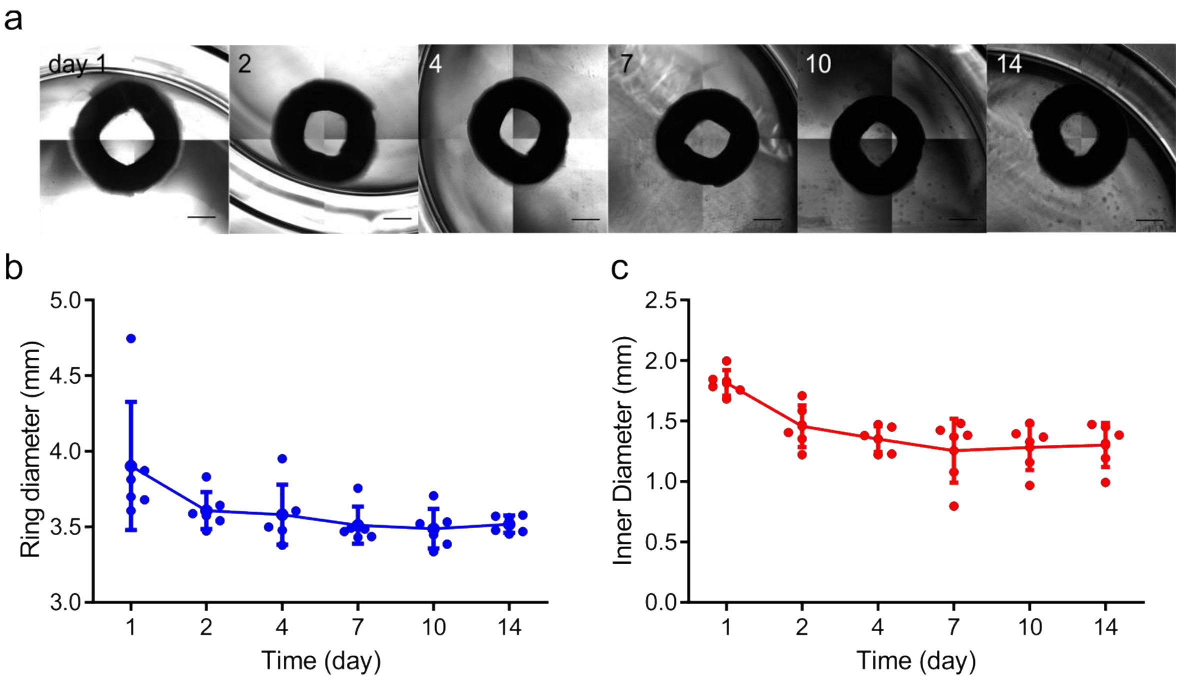

Supplementary Figure 4: Morphological Changes of Fibroblast Tissue Rings. (a) The morphology of fibroblast tissue rings after extraction retained the ring shape with consistent (b) outer ring diameter and (c) inner diameter. Scale bar: 1 mm.

Supplementary Table 1: The primary antibodies used for immunofluorescent microscopy in this study.

**Antibodies**	**Markers**	**Vendors**	**Cat #**	**Dilution**
NANOG	hiPSC	Life Technologies	23D2-3C6	1:200
SOX10	NCSC	R & D Systems	MAB2864-SP	1:50
FOXD3	NCSC	R & D Systems	AF5090-SP	1:20
NGFR	NCSC	R & D Systems	MAB367-SP	1:50
CD44	MSC	Bio-Rad Laboratories	MCA2726	1:100
CD73	MSC	Abcam	ab133582	1:50
CD90	MSC	Abcam	ab181469	1:200
CD105	MSC	Life Technologies	MA5-11854	1:100
CD106	MSC	Bio-Rad Laboratories	MCA2297	1:100
CD144	MSC	Sigma Aldrich	V1514	1:200
CD166	MSC	Bio-Rad Laboratories	MCA1926	1:100
